# 2-(5,6-Dibromo-7-methyl-3*H*-imidazo[4,5-*b*]pyridin-2-yl)phenol

**DOI:** 10.1107/S1600536810045277

**Published:** 2010-11-13

**Authors:** Haixia Wang

**Affiliations:** a Department of Chemistry and Environmental Science, Henan Normal University, Xinxiang 453007, People’s Republic of China

## Abstract

In the title compound, C_13_H_9_Br_2_N_3_O, the mol­ecular skeleton, influenced by an intra­molecular O—H⋯N hydrogen bond, is roughly planar, with a mean deviation of 0.033 Å. In the crystal, inter­molecular N—H⋯O hydrogen bonds link the mol­ecules into chains propagating in [100]. Weak inter­molecular π–π inter­actions [centroid–centroid distances = 3.760 (3) and 3.723 (3) Å] further consolidate the packing.

## Related literature

For background to the use of imidazole and its derivatives in transition metal complexes, see: Huang *et al.* (2004 ▶). For related structures, see: Eltayeb *et al.* (2009 ▶); Xiao *et al.* (2009 ▶); Elerman & Kabak (1997 ▶).
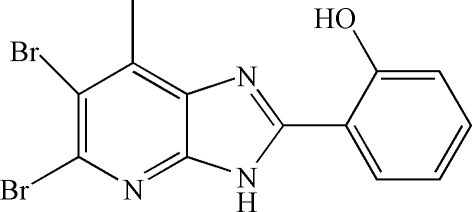


## Experimental

### 

#### Crystal data

C_13_H_9_Br_2_N_3_O
*M* *_r_* = 383.05
Orthorhombic, 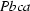

*a* = 13.181 (5) Å
*b* = 8.494 (3) Å
*c* = 22.692 (8) Å
*V* = 2540.5 (16) Å^3^
*Z* = 8
Mo *K*α radiation
μ = 6.38 mm^−1^
*T* = 293 K
0.31 × 0.28 × 0.24 mm

#### Data collection

Bruker APEXII CCD area-detector diffractometer
Absorption correction: multi-scan (*SADABS*; Sheldrick, 2008*a* ▶) *T*_min_ = 0.243, *T*_max_ = 0.310
11656 measured reflections
2234 independent reflections
1706 reflections with *I* > 2σ(*I*)
*R*_int_ = 0.041

#### Refinement

*R*[*F* ^2^ > 2σ(*F* ^2^)] = 0.043
*wR*(*F* ^2^) = 0.138
*S* = 1.08
2234 reflections
173 parameters
H-atom parameters constrained
Δρ_max_ = 0.63 e Å^−3^
Δρ_min_ = −0.88 e Å^−3^

### 

Data collection: *APEX2* (Bruker, 2004 ▶); cell refinement: *SAINT-Plus* (Bruker, 2001 ▶); data reduction: *SAINT-Plus*; program(s) used to solve structure: *SHELXS97* (Sheldrick, 2008*b* ▶); program(s) used to refine structure: *SHELXL97* (Sheldrick, 2008*b* ▶); molecular graphics: *SHELXTL* (Sheldrick, 2008*b* ▶); software used to prepare material for publication: *SHELXTL*.

## Supplementary Material

Crystal structure: contains datablocks I, global. DOI: 10.1107/S1600536810045277/cv2784sup1.cif

Structure factors: contains datablocks I. DOI: 10.1107/S1600536810045277/cv2784Isup2.hkl

Additional supplementary materials:  crystallographic information; 3D view; checkCIF report

## Figures and Tables

**Table 1:** Hydrogen-bond geometry (Å, °)

*D*—H⋯*A*	*D*—H	H⋯*A*	*D*⋯*A*	*D*—H⋯*A*
N1—H1*A*⋯O1^i^	0.95	1.90	2.839 (6)	171
O1—H1⋯N2	0.82	1.84	2.573 (6)	149

Symmetry code: (i) 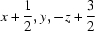.

**Fig. 1.**
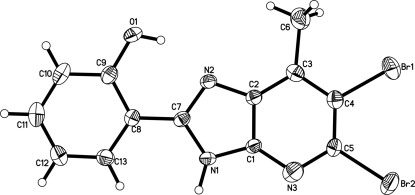
The molecular structure of the title compound showing the atom-labelling scheme and 30% probabilty displament ellipsoids.

## supplementary crystallographic information

### Comment

Due to excellent coordination abilities the imidazole and its derivatives have already been introduced into the transition metal complexes (Huang *et al.*, 2004). Herewith we present the title compound (I) - a new imidazole derivative.

In (I) (Fig. 1), intramolecular O—H···N hydrogen bond (Table 2) influence the molecular conformation, so all non-H atoms are nearly coplanar with the mean deviation of 0.033 Å. The dihedral angle between the 5,6-dibromo-7-methyl-3*H*-imidazo[4,5-*b*]pyridine plane and the phenol plane is 2.1 (2) °. The bond lengths and angles are normal and comparable to those observed in the reported benzimidazole compounds (Xiao *et al.*, 2009; Eltayeb *et al.*, 2009; Elerman & Kabak 1997).

In the crystal structure, intermolecular N—H···O hydrogen bonds (Table 2) link the molecules into chains propagated in direction [100]. Weak intermolecular π—π interactions (Table 1) consolidate further the crystal packing.

### Experimental

The title compound was synthesized by the reaction of 4-methyl-2,3-diamino-5,6-dibromopyridine and 2-hydroxybenzaldehyde with the ratio 1:1 in ethanol. After the mixture was refluxed sevral hours, the resulting clear yellow solution was allowed to evaporate slowly in air, and orange-yellow block-like crystals suitable for X-ray diffraction were obtained with a yield 47% about ten days later.

### Refinement

All the H atoms bonded to the C atoms were placed using the HFIX commands in *SHELXL-97* with C—H distances of 0.93 and 0.96 Å, and were refined as riding, with *U*_iso_(H) = 1.2-1.5*U*_eq_(C). H atoms bonded to O and N atoms were found from difference Fourier maps with the bond lengths restrained to 0.82 and 0.96 Å, respectively, and were refined as riding, with *U*_iso_(H) = 1.5*U*_eq_(O) and *U*_iso_(H) = 1.2*U*_eq_(N).

### Crystal data

**Table:**

C_13_H_9_Br_2_N_3_O	*F*(000) = 1488
*M**_r_* = 383.05	*D*_x_ = 2.003 Mg m^−^^3^
Orthorhombic, *P**b**c**a*	Mo *K*α radiation, λ = 0.71073 Å
Hall symbol: -P 2ac 2ab	Cell parameters from 1452 reflections
*a* = 13.181 (5) Å	θ = 2.9–24.6°
*b* = 8.494 (3) Å	µ = 6.38 mm^−^^1^
*c* = 22.692 (8) Å	*T* = 293 K
*V* = 2540.5 (16) Å^3^	Block, orange–yellow
*Z* = 8	0.31 × 0.28 × 0.24 mm

### Data collection

**Table:**

Bruker APEXII CCD area-detector diffractometer	2234 independent reflections
Radiation source: fine-focus sealed tube	1706 reflections with *I* > 2σ(*I*)
graphite	*R*_int_ = 0.041
φ and ω scans	θ_max_ = 25.0°, θ_min_ = 1.8°
Absorption correction: multi-scan (*SADABS*; Sheldrick, 2008*a*)	*h* = −15→15
*T*_min_ = 0.243, *T*_max_ = 0.310	*k* = −9→10
11656 measured reflections	*l* = −24→26

### Refinement

**Table:**

Refinement on *F*^2^	Primary atom site location: structure-invariant direct methods
Least-squares matrix: full	Secondary atom site location: difference Fourier map
*R*[*F*^2^ > 2σ(*F*^2^)] = 0.043	Hydrogen site location: inferred from neighbouring sites
*wR*(*F*^2^) = 0.138	H-atom parameters constrained
*S* = 1.08	*w* = 1/[σ^2^(*F*_o_^2^) + (0.0692*P*)^2^ + 6.558*P*] where *P* = (*F*_o_^2^ + 2*F*_c_^2^)/3
2234 reflections	(Δ/σ)_max_ = 0.001
173 parameters	Δρ_max_ = 0.63 e Å^−^^3^
0 restraints	Δρ_min_ = −0.88 e Å^−^^3^

### Special details

**Table:**

Geometry. All e.s.d.'s (except the e.s.d. in the dihedral angle between two l.s. planes) are estimated using the full covariance matrix. The cell e.s.d.'s are taken into account individually in the estimation of e.s.d.'s in distances, angles and torsion angles; correlations between e.s.d.'s in cell parameters are only used when they are defined by crystal symmetry. An approximate (isotropic) treatment of cell e.s.d.'s is used for estimating e.s.d.'s involving l.s. planes.
Refinement. Refinement of *F*^2^ against ALL reflections. The weighted *R*-factor *wR* and goodness of fit *S* are based on *F*^2^, conventional *R*-factors *R* are based on *F*, with *F* set to zero for negative *F*^2^. The threshold expression of *F*^2^ > σ(*F*^2^) is used only for calculating *R*-factors(gt) *etc*. and is not relevant to the choice of reflections for refinement. *R*-factors based on *F*^2^ are statistically about twice as large as those based on *F*, and *R*- factors based on ALL data will be even larger.

### Fractional atomic coordinates and isotropic or equivalent isotropic displacement parameters (Å^2^)

**Table:**

	*x*	*y*	*z*	*U*_iso_*/*U*_eq_	
Br1	0.27968 (6)	1.22305 (9)	0.48970 (3)	0.0618 (3)	
Br2	0.51701 (6)	1.19032 (9)	0.54180 (3)	0.0669 (3)	
O1	0.0310 (3)	0.7707 (6)	0.73898 (19)	0.0541 (12)	
H1	0.0614	0.8111	0.7113	0.081*	
N1	0.3413 (3)	0.8522 (5)	0.7102 (2)	0.0380 (10)	
H1A	0.4011	0.8215	0.7306	0.046*	
N2	0.1806 (3)	0.8887 (5)	0.68102 (19)	0.0365 (10)	
N3	0.4280 (4)	1.0090 (7)	0.6313 (2)	0.0615 (15)	
C1	0.3468 (4)	0.9445 (6)	0.6601 (2)	0.0348 (12)	
C2	0.2452 (4)	0.9659 (6)	0.6416 (2)	0.0350 (12)	
C3	0.2226 (4)	1.0517 (7)	0.5909 (2)	0.0432 (13)	
C4	0.3060 (4)	1.1146 (7)	0.5612 (2)	0.0414 (13)	
C5	0.4049 (4)	1.0950 (6)	0.5811 (2)	0.0400 (13)	
C6	0.1127 (5)	1.0806 (9)	0.5643 (3)	0.0680 (19)	
H6A	0.0923	1.1873	0.5717	0.102*	
H6B	0.0653	1.0098	0.5825	0.102*	
H6C	0.1140	1.0621	0.5226	0.102*	
C7	0.2410 (4)	0.8221 (6)	0.7213 (2)	0.0357 (12)	
C8	0.2041 (4)	0.7275 (6)	0.7696 (2)	0.0354 (12)	
C9	0.0988 (5)	0.7064 (7)	0.7768 (3)	0.0431 (13)	
C10	0.0636 (5)	0.6169 (8)	0.8237 (3)	0.0578 (17)	
H10	−0.0057	0.6022	0.8288	0.069*	
C11	0.1315 (5)	0.5488 (8)	0.8632 (3)	0.0604 (18)	
H11	0.1074	0.4895	0.8947	0.072*	
C12	0.2329 (5)	0.5690 (8)	0.8558 (3)	0.0536 (16)	
H12	0.2779	0.5226	0.8821	0.064*	
C13	0.2693 (4)	0.6560 (7)	0.8104 (3)	0.0453 (14)	
H13	0.3390	0.6686	0.8063	0.054*	

### Atomic displacement parameters (Å^2^)

**Table:**

	*U*^11^	*U*^22^	*U*^33^	*U*^12^	*U*^13^	*U*^23^
Br1	0.0851 (6)	0.0621 (5)	0.0383 (4)	0.0019 (4)	−0.0058 (3)	0.0082 (3)
Br2	0.0640 (5)	0.0757 (5)	0.0609 (5)	−0.0265 (4)	0.0124 (3)	0.0054 (4)
O1	0.029 (2)	0.079 (3)	0.054 (3)	0.0054 (19)	0.0065 (19)	0.007 (2)
N1	0.027 (2)	0.048 (3)	0.039 (3)	−0.0008 (19)	−0.0055 (19)	0.001 (2)
N2	0.028 (2)	0.045 (3)	0.037 (3)	0.0009 (18)	−0.0012 (19)	−0.002 (2)
N3	0.056 (3)	0.068 (4)	0.060 (4)	−0.006 (3)	0.003 (3)	−0.005 (3)
C1	0.029 (3)	0.043 (3)	0.033 (3)	−0.004 (2)	0.000 (2)	−0.003 (2)
C2	0.033 (3)	0.040 (3)	0.032 (3)	−0.001 (2)	0.000 (2)	−0.004 (2)
C3	0.048 (3)	0.046 (3)	0.036 (3)	0.007 (3)	−0.002 (3)	−0.005 (3)
C4	0.050 (3)	0.041 (3)	0.034 (3)	−0.002 (3)	0.000 (3)	−0.003 (2)
C5	0.045 (3)	0.040 (3)	0.036 (3)	−0.007 (2)	0.006 (3)	0.000 (2)
C6	0.071 (5)	0.076 (5)	0.057 (4)	0.012 (4)	0.011 (4)	0.010 (4)
C7	0.031 (3)	0.040 (3)	0.036 (3)	−0.001 (2)	0.004 (2)	−0.007 (2)
C8	0.038 (3)	0.034 (3)	0.034 (3)	−0.002 (2)	0.004 (2)	−0.004 (2)
C9	0.044 (3)	0.044 (3)	0.041 (3)	0.001 (3)	0.009 (3)	−0.006 (3)
C10	0.054 (4)	0.061 (4)	0.059 (4)	−0.008 (3)	0.023 (3)	−0.001 (3)
C11	0.082 (5)	0.051 (4)	0.048 (4)	−0.004 (3)	0.018 (3)	0.006 (3)
C12	0.066 (4)	0.052 (4)	0.043 (4)	0.001 (3)	−0.004 (3)	0.008 (3)
C13	0.042 (3)	0.047 (3)	0.047 (4)	−0.001 (3)	−0.006 (3)	0.003 (3)

### Geometric parameters (Å, °)

**Table:**

Br1—C4	1.897 (6)	C4—C5	1.389 (8)
Br2—C5	1.907 (5)	C6—H6A	0.9600
O1—C9	1.354 (7)	C6—H6B	0.9600
O1—H1	0.8200	C6—H6C	0.9600
N1—C7	1.369 (7)	C7—C8	1.442 (8)
N1—C1	1.382 (7)	C8—C13	1.402 (8)
N1—H1A	0.9504	C8—C9	1.408 (8)
N2—C7	1.338 (7)	C9—C10	1.388 (8)
N2—C2	1.398 (7)	C10—C11	1.392 (9)
N3—C1	1.370 (7)	C10—H10	0.9300
N3—C5	1.387 (8)	C11—C12	1.358 (9)
C1—C2	1.415 (7)	C11—H11	0.9300
C2—C3	1.394 (8)	C12—C13	1.355 (8)
C3—C4	1.396 (8)	C12—H12	0.9300
C3—C6	1.589 (9)	C13—H13	0.9300
			
Cg1···Cg2^i^	3.760 (3)	Cg1···Cg3^ii^	3.723 (3)
			
C9—O1—H1	109.5	C3—C6—H6C	109.5
C7—N1—C1	107.9 (4)	H6A—C6—H6C	109.5
C7—N1—H1A	131.3	H6B—C6—H6C	109.5
C1—N1—H1A	120.8	N2—C7—N1	111.7 (5)
C7—N2—C2	105.9 (4)	N2—C7—C8	123.6 (5)
C1—N3—C5	115.6 (5)	N1—C7—C8	124.7 (5)
N3—C1—N1	131.4 (5)	C13—C8—C9	118.1 (5)
N3—C1—C2	123.1 (5)	C13—C8—C7	122.4 (5)
N1—C1—C2	105.6 (4)	C9—C8—C7	119.5 (5)
C3—C2—N2	130.1 (5)	O1—C9—C10	119.1 (6)
C3—C2—C1	121.0 (5)	O1—C9—C8	121.6 (5)
N2—C2—C1	109.0 (5)	C10—C9—C8	119.3 (6)
C2—C3—C4	115.5 (5)	C9—C10—C11	120.4 (6)
C2—C3—C6	126.1 (5)	C9—C10—H10	119.8
C4—C3—C6	118.4 (5)	C11—C10—H10	119.8
C5—C4—C3	122.4 (5)	C12—C11—C10	120.0 (6)
C5—C4—Br1	120.5 (4)	C12—C11—H11	120.0
C3—C4—Br1	117.1 (4)	C10—C11—H11	120.0
N3—C5—C4	122.5 (5)	C13—C12—C11	120.8 (6)
N3—C5—Br2	115.9 (4)	C13—C12—H12	119.6
C4—C5—Br2	121.6 (4)	C11—C12—H12	119.6
C3—C6—H6A	109.5	C12—C13—C8	121.5 (5)
C3—C6—H6B	109.5	C12—C13—H13	119.3
H6A—C6—H6B	109.5	C8—C13—H13	119.3

Symmetry codes: (i) −*x*+1/2, *y*+1/2, *z*; (ii) −*x*+1/2, *y*−1/2, *z*.

### Hydrogen-bond geometry (Å, °)

**Table:**

*D*—H···*A*	*D*—H	H···*A*	*D*···*A*	*D*—H···*A*
N1—H1A···O1^iii^	0.95	1.90	2.839 (6)	171
O1—H1···N2	0.82	1.84	2.573 (6)	149

Symmetry codes: (iii) *x*+1/2, *y*, −*z*+3/2.
